# Circulating Small Extracellular Vesicles Reflect the Severity of Myocardial Damage in STEMI Patients

**DOI:** 10.3390/biom13101470

**Published:** 2023-09-29

**Authors:** Marta Zarà, Andrea Baggiano, Patrizia Amadio, Jeness Campodonico, Sebastiano Gili, Andrea Annoni, Gianluca De Dona, Maria Ludovica Carerj, Francesco Cilia, Alberto Formenti, Laura Fusini, Cristina Banfi, Paola Gripari, Calogero Claudio Tedesco, Maria Elisabetta Mancini, Mattia Chiesa, Riccardo Maragna, Francesca Marchetti, Marco Penso, Luigi Tassetti, Alessandra Volpe, Alice Bonomi, Giancarlo Marenzi, Gianluca Pontone, Silvia Stella Barbieri

**Affiliations:** 1 Centro Cardiologico Monzino IRCCS, 20138 Milan, Italy; marta.zara@cardiologicomonzino.it (M.Z.); andrea.baggiano@cardiologicomonzino.it (A.B.); giancarlo.marenzi@cardiologicomonzino.it (G.M.);; 2 Department of Clinical Sciences and Community Health, University of Milan, 20122 Milan, Italy; 3 Department of Electronics, Information and Bioengineering, Politecnico di Milano, 20156 Milan, Italy; 4 Department of Biomedical, Surgical and Dental Sciences, University of Milan, 20122 Milan, Italy

**Keywords:** small extracellular vesicles, cardiac magnetic resonance, microvascular obstruction, myocardial infarction, platelets

## Abstract

Circulating small extracellular vesicles (sEVs) contribute to inflammation, coagulation and vascular injury, and have great potential as diagnostic markers of disease. The ability of sEVs to reflect myocardial damage assessed by Cardiac Magnetic Resonance (CMR) in ST-segment elevation myocardial infarction (STEMI) is unknown. To fill this gap, plasma sEVs were isolated from 42 STEMI patients treated by primary percutaneous coronary intervention (pPCI) and evaluated by CMR between days 3 and 6. Nanoparticle tracking analysis showed that sEVs were greater in patients with anterior STEMI (*p* = 0.0001), with the culprit lesion located in LAD (*p* = 0.045), and in those who underwent late revascularization (*p* = 0.038). A smaller sEV size was observed in patients with a low myocardial salvage index (MSI, *p* = 0.014). Patients with microvascular obstruction (MVO) had smaller sEVs (*p* < 0.002) and lower expression of the platelet marker CD41–CD61 (*p* = 0.039). sEV size and CD41–CD61 expression were independent predictors of MVO/MSI (OR [95% CI]: 0.93 [0.87–0.98] and 0.04 [0–0.61], respectively). In conclusion, we provide evidence that the CD41–CD61 expression in sEVs reflects the CMR-assessed ischemic damage after STEMI. This finding paves the way for the development of a new strategy for the timely identification of high-risk patients and their treatment optimization.

## 1. Introduction

Acute myocardial infarction (AMI) is the leading cause of mortality and morbidity worldwide. ST-elevation myocardial infarction (STEMI) is the worst clinical presentation of AMI, and although timely reperfusion by primary percutaneous coronary intervention (pPCI) has significantly reduced the extent of myocardial tissue damage, the prognosis of these patients is still not satisfactory. Indeed, reperfusion therapy itself may lead to additional myocardial tissue damage, known as ischemia-reperfusion injury. 

The extent of edema, infarct dimensions, myocardial salvage index (MSI), myocardial hemorrhage (MH), and microvascular obstruction (MVO) are major components of reperfusion injury and have a strong prognostic value in predicting functional recovery after STEMI. In this context, cardiac magnetic resonance (CMR) plays a crucial role given its ability to non-invasively depict and quantify the diverse components of ischemic damage with high precision and reproducibility [1]. CMR is, therefore, the gold standard imaging technique for evaluating myocardial injury after STEMI but it is not always applicable, due to resource/availability reasons and to patients’ contraindications (e.g., contrast media reactions, not compatible implanted devices, claustrophobia) [2]. Therefore, the development and validation of alternative/additional tools are needed to accommodate those patients who are unfavorable candidates for CMR imaging. 

Small extracellular vesicles (sEVs), also known as “exosomes”, are lipid vesicles (30–150 nm in diameter) originating from multivesicular bodies, that are actively released by different cell types and are detectable in various body fluids, including plasma. Mainly known for their central role in local/distant intercellular communication, the growing knowledge about sEVs has highlighted their potential as a rich source of biomarkers for several diseases [3,4]. We have recently shown that the number and size of plasma sEVs are higher in acute STEMI patients compared to chronic coronary syndrome (CCS) [5]. Moreover, STEMI sEVs are characterized by a greater expression of platelet markers [5], suggesting a critical role for platelet-derived sEVs in AMI. Interestingly, a lower number of platelet-derived extracellular vesicles is associated with adverse cardiac remodeling [6], a frequent complication of STEMI associated with worse cardiac function and poor prognosis. The role of platelets in thrombus formation as well as their involvement in myocardial damage in AMI patients is well known [7,8,9]. Similarly, several studies have investigated the contribution of circulating microvesicles (MVs), particularly those of platelet origin, in myocardial infarction both as a therapeutic target and biomarker [10,11,12,13]. However, sEVs have usually not been included in previous analyses due to their small size and limited resolution of conventional equipment, so little information is available on the role of sEVs in this clinical scenario. The aim of this proof-of-concept study was to determine whether the profile of plasma sEVs, particularly platelet-derived, reflects CMR features/characteristics in the clinical context of STEMI after pPCI.

## 2. Materials and Methods

An expanded discussion of materials and methods used is available in the online-only Data Supplement.

### 2.1. Study Population and Plasma Sample Collection

One-hundred forty-five consecutive patients with STEMI, referred to our hospital were prospectively screened to identify those meeting the following inclusion criteria: (1) chest pain suggestive of myocardial ischemia lasting >30 min, (2) ST-segment elevation > 0.1 mV in ≥2 limb leads or >0.2 mV in ≥2 contiguous precordial leads, or presumed new left bundle branch block, and (3) successful treatment with pPCI within 12 h from symptom onset. Exclusion criteria were prior myocardial infarction or revascularization, time to pPCI > 12 h, atrial fibrillation, Killip class > III, renal failure (glomerular filtration < 30 mL/min), claustrophobia or other contraindications to CMR. Each patient underwent blood collection and clinically indicated CMR before discharge. 

The study took advantage of EDTA plasma samples prepared from a cohort of 145 consecutive STEMI patients enrolled between January 2015 and May 2017 at the Centro Cardiologico Monzino IRCCS [14]. The study complied with the Declaration of Helsinki. The ethics committee approved the research protocol (R114/14-CCM 125), and informed consent was obtained from each subject. The plasma samples have been also used for other studies; therefore, only 42 out of 145 plasma samples had a sufficient volume to perform the analyses required for this work.

In our study population, we considered revascularization performed < 3 h after symptom onset to be “early”, and revascularizations ≥ 3 h to be “late”.

### 2.2. CMR Protocol and Analysis

CMR exams were performed as previously described [14]. Briefly, all patients were studied with a 1.5-T scanner (Discovery MR450, GE Healthcare, Milwaukee, WI, USA) within one week after pPCI. All CMR data were transferred to a dedicated workstation (Circle Cardiovascular Imaging: CVI 42, Calgary, AB, Canada) and were evaluated by two expert readers with more than 5 years of experience in CMR performance and analysis. The scan protocol was performed according to the guidelines of the Society of Cardiovascular Magnetic Resonance [15]. The following morpho-functional parameters were calculated from the short-axis cine images: indexed left and right end-diastolic volume (LVEDVi and RVEDVi), indexed left and right end-systolic volume (LVESVi and RVESVi), left and right ejection fraction (LVEF and RVEF), LV mass. Breath-hold black-blood T2-weighted short inversion-time inversion-recovery fast spin-echo (T2-weighted) imaging was performed with the same prescription of cine CMR images. Myocardium with a signal intensity > 2 SD above the mean signal intensity of remote non-infarcted myocardium was considered the area at risk (AAR) and it was measured as absolute mass and as a percentage of entire LV mass [16]. Furthermore, T2-weighted images were used to define MH as a hypoenhanced region within the AAR. After the complete acquisition of non-contrast images, 0.1 mmol/Kg of Gadolinium-BOPTA (Multihance, Bracco, Milan, Italy) was administered at a flow rate of 3 mL/s followed by 20 mL of saline flush. Ten minutes after the injection of contrast material, breath-hold contrast-enhanced segmented T1-weighted inversion-recovery gradient-echo sequences were acquired in order to identify late gadolinium enhancement (LGE) [17]. On post-contrast imaging, LGE was automatically marked by dedicated software with a threshold approach considered to be present if the signal intensity of the hyperenhanced myocardium was >5 SD above the mean signal intensity of the remote myocardium [18]. Moreover, MVO was defined as the hypoenhanced region within the LGE area and was quantified by careful manual delineation of this hypoenhanced region with the same dedicated software used for LGE quantification. Finally, both LGE and MVO were reported as absolute mass and as a percentage of the entire LV mass.

### 2.3. Nanoparticle Tracking Analysis (NTA)

Concentration and size distribution of sEVs samples were measured with NanoSight (NS300) (Malvern Panalytical Ltd., Malvern, UK ) equipped with NTA software (version 3.4; Malvern Panalytical Ltd., Malvern, UK) s previously described [5]. Briefly, Plasma-derived sEVs were isolated from EDTA plasma using Invitrogen™ Total Exosome Isolation Kit (from plasma) (Thermo Fisher Scientific, Waltham, MA, USA) as previously described [6]. Briefly, 250 µL of plasma was centrifuged at 10,000× *g* for 20 min. The clarified plasma was diluted with phosphate-buffered saline (PBS) and the Exosome Precipitation Reagent was added, incubated at room temperature for 10 min and finally centrifuged at 10,000× *g* for 5 min. The supernatant was carefully discarded, and the pellet of sEVs was resuspended in PBS. The concentration and size distribution of sEVs samples were measured with NanoSight (NS300) (Malvern Panalytical Ltd.) equipped with NTA software (version 3.4; Malvern Panalytical Ltd., Malvern, UK). All samples were diluted to the appropriate concentration and five videos of 60 s were recorded for each sample and analyzed under constant settings using NTA software. The settings were established according to the manufacturer’s software manual (NanoSight NS300 User Manual, MAN0541-01-EN-00, 2017). 

### 2.4. Surface Epitope Analysis

sEVs surface epitopes were analyzed by the MACSPlex Exosome Kit (Miltenyi Biotec, Washington, DC, USA), following manufacturer instructions. This kit allows detection of 37 exosomal surface epitopes (CD1c, CD2, CD3, CD4, CD8, CD9, CD11c, CD14, CD19, CD20, CD24, CD25, CD29, CD31, CD40, CD41b, CD42a, CD44, CD45, CD49e, CD56, CD62P, CD63, CD69, CD81, CD86, CD105, CD133/1, CD142, CD146, CD209, CD326, HLA-ABC, HLA-DRDPDQ, MCSP, ROR1 and SSEA-4) plus two isotype controls (mIgG1 and REA). 

Briefly, sEVs were isolated from plasma with Exosome Isolation Kit CD63, human (Miltenyi Biotec), following manufacturer instructions. Samples were then incubated with the antibody-coated MACSPlex Exosome Capture Beads, and they were labeled with the MACSPlex Exosome Detection Reagents (APC-conjugated anti-CD9, anti-CD63, and anti-CD81 detection antibody). Sandwich complexes were analyzed based on their fluorescence characteristics in an Attune™ NxT Flow Cytometer (Invitrogen™). Median fluorescence signal intensity (MFI) for all 39 capture bead subsets was background corrected by subtracting respective MFI values from the negative control.

### 2.5. Pathways Analysis and Network Graphs Generation

Functional analysis was performed using the Cytoscape (v. 3.9.1) [19] plug-in ClueGO (v. 2.5.8) [20] which estimates the pathways enrichment score on a pre-selected set of genes, exploiting a two-sided hypergeometric test. The Reactome pathway database has been selected as a reference [21]. Pathways with less than four associated genes from the uploaded gene list were discarded. Functionally related terms were grouped by setting a similarity threshold of a kappa score of 0.4. Pathways with an associated *p*-value (*p*) < 0.01 were deemed as significant. All network graphs were drawn by Cytoscape built-in functions. The relation between antigens and the associated genes is reported in Supplementary Table S3.

### 2.6. ELISA

Plasma-derived sEVs were isolated from EDTA plasma using Invitrogen™ Total Exosome Isolation Kit (from plasma) (Thermo Fisher Scientific) and sEV expression of CD41–CD61 was quantified by Human ELISA kit (Abcam, Cambridge, UK) according to supplier’s instructions.

### 2.7. Statistical Analysis

Statistical analysis was performed using the GraphPad Prism 8.0 Software (GraphPad Software Inc., La Jolla, CA, USA) and SAS statistical software (v.9.4). The distribution of continuous variables was assessed by visual inspection of frequency histograms and with the use of the Shapiro–Wilk test. Continuous variables were expressed as mean ± standard deviation (SD) and were compared using the t-test for independent samples. The associations between variables of sEV profile and reperfusion myocardial injury were analyzed by univariate and multivariate logistic models. Three models were applied: model 1: unadjusted, model 2: adjusted to STEMI site; model 3: a model including all the three sEV profile variables and STEMI site. A receiver-operating characteristic (ROC) was performed to evaluate the accuracy of the sEV profile to predict reperfusion myocardial injury. All tests were two-tailed, and a value of *p* < 0.05 was considered to be statistically significant. 

## 3. Results

### 3.1. Patient Characteristics

Table 1 and Table 2 summarized the baseline and CMR characteristics of our study population. The mean age was 63.1 ± 9.7 years, the majority of patients were males (73.8%), nineteen (45.2%) patients had an anterior STEMI and the mean LVEF at CMR was 51 ± 11.2%. CMR showed an AAR involving 22.8 ± 18.3% of LV mass, with an LGE extent of 14.3 ± 17.6% of LV mass. MVO occurred in 20 (47.6%) patients.

### 3.2. NTA of Plasma sEVs

A thorough evaluation of circulating plasma sEVs was performed by assessing their number, dimension and specific antigens’ expression. The characteristics of sEVs were then analyzed according to typical clinical features (including the site of STEMI, the location of culprit lesion, time to revascularization, and troponin peak) and parameters assessed by CMR performed in the first few days post-pPCI (MVO and MSI) (Tables S1 and S2), all parameters related to the extent of cardiac damage and strongly prognostic. 

**Figure 1:**
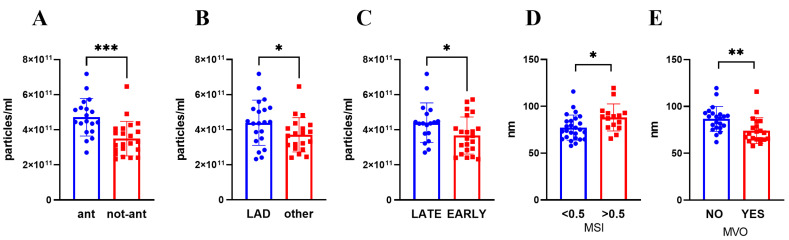
sEVs number (**A**–**C**) and size (**D**,**E**) were evaluated by NTA after isolation from plasma collected 3–5 days post STEMI. Patients were divided according to (**A**) STEMI site, (**B**) culprit lesion artery, (**C**) presentation time, (**D**) MSI (low <0.5; high >0.5), (**E**) MVO presence. Mean ± SD are reported. * *p* < 0.05; ** *p* < 0.01; *** *p* < 0.005. ant: anterior STEMI, non-ant: non-anterior STEMI, LAD: Left anterior descending artery, MVO: microvascular obstruction, MSI: myocardial salvage index.

Focusing on STEMI location, anterior myocardial infarction displayed a higher number of circulating sEVs (*p* = 0.0001) but no differences in their dimension (Figure 1, Table S1). In line with this, an elevated level of sEVs was detected when the culprit lesion was located in the left anterior descending artery (LAD) (*p* = 0.045, Figure 1, Table S1). The level of circulating sEVs, but not their dimension, is influenced also by the time to revascularization, with a higher number of sEVs in patients who underwent a late revascularization (≥3 h after symptom onset) (*p* = 0.038) (Figure 1, Table S1).

By contrast, sEV dimension correlated negatively with LGE mass and MVO extent (Table S2). Moreover, by sub-diving patients according to the presence of MVO and MSI value, we found that a smaller sEV dimension was associated with the presence of MVO (*p* < 0.002) and to lower MSI value (≤0.5) (*p* = 0.014) (Figure 1, Table S1). Interestingly, the number and the dimension of circulating sEVs were neither correlated to the troponin peak nor CMR-assessed EF (Table S2).

### 3.3. sEVs Surface Antigen Expression and Pathway Analysis

**Figure 2:**
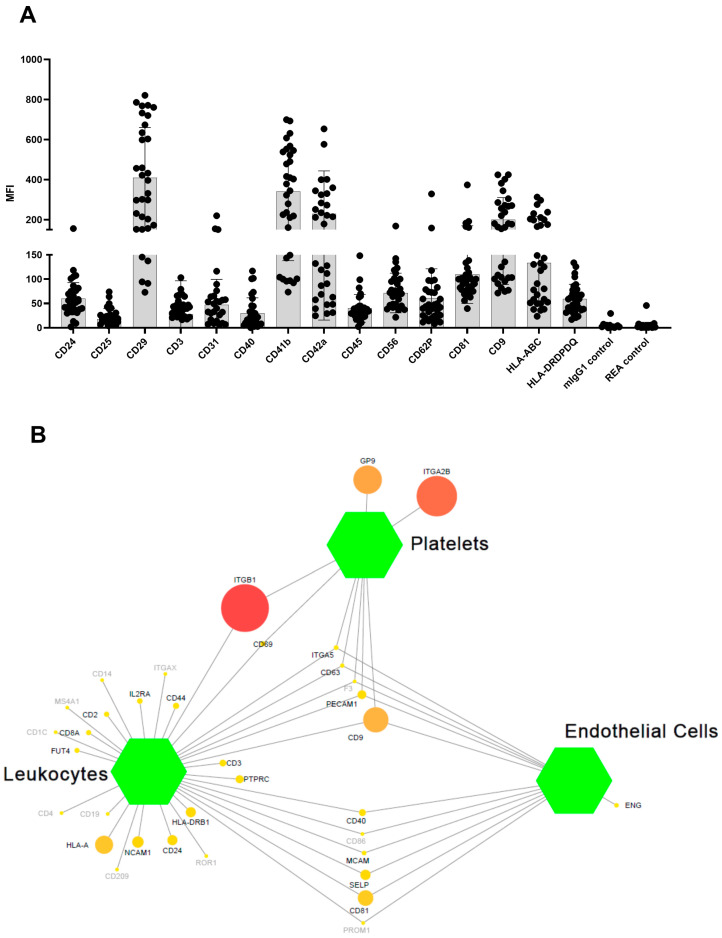
Surface Epitope characterization of plasma sEVs. (**A**) Dot plot showing the MFI distribution for the reported antigens. (**B**) Network graph mapping the correspondence between the investigated antigens (circle node) and specific cell types (green hexagons); the color gradient and the node size represent the MFI expression from low (yellow and small nodes) to high (red and big nodes) level. Gray nodes denote not expressed antigens.

The analysis of sEVs by MACSPlex revealed surface marker profiles characterized by strong signals for the exosome-associated markers (CD9 and CD81). The data additionally indicated the presence of other markers, with the highest intensity shown for platelet-derived antigens (CD29, CD41b, CD42a). On the other hand, antigens typically over-represented in leukocytes and endothelial cells have moderate-to-low expression levels (Figure 2A). Using pathway analysis, which allows inferring the functional role of specific genomic signatures, we found that the overrepresented antigens in our study are mainly involved in pathways related to platelet activation, immune response, and cell-cell interactions (Figure 2B and Figure S1). More details on pathways, such as term description, *p*-value and the name of associated antigens are shown in Tables S3 and S4. 

**Figure 3:**
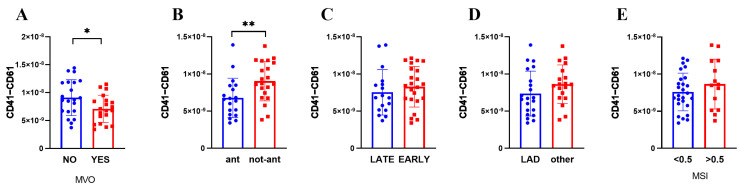
sEV expression of platelet marker CD41–CD61. Patients were divided according to (**A**) MVO presence, (**B**) STEMI site, (**C**) presentation time, (**D**) culprit lesion artery, (**E**) MSI (low <0.5; high >0.5). Mean ± SD are reported. * *p* < 0.05; ** *p* < 0.01.

The expression of platelet-specific markers was further investigated by an ELISA assay, focusing on the platelet-specific marker CD41–CD61 (also known as Glycoprotein IIbIIIa). We found a lower expression of CD41–CD61 when AMI is located in the anterior wall (*p* = 0.004) and in patients with MVO (*p* = 0.039) (Figure 3). Interestingly, CD41–CD61 expression correlated negatively with extent of MVO (r = −0.40; *p* = 0.01) and positively with EF (r = 0.31; *p* = 0.04) (Table S5). Importantly, none of the sEV characteristics were different between sexes (Table S6) or correlated with age (Table S7).

### 3.4. Association between Variables of sEV Profile and Myocardial Injury

Finally, univariate and multivariate logistic models were used to thoroughly evaluate the association between sEV profile and reperfusion myocardial injury, here defined as the presence of low MSI or MVO. To obtain a complete and careful overview three models were applied: model 1, which was unadjusted; model 2, which was adjusted to the STEMI site; model 3, which included all the three sEV profile variables and STEMI site.

We found a significant association of MVO/MSI with sEV size and CD41–CD61 expression both in model 1 (unadjusted) and in model 2 (adjusted to STEMI site). Finally, model 3 actually confirmed that sEV size and CD41–CD61 were independent predictors of MVO/MSI. In contrast, plasma sEV concentration was not associated with MVO/MSI (Table 3). 

When the two parameters (MVO and MSI) were analyzed separately (Table 3), the relationship between MVO and sEV size or CD41–CD61 was confirmed in all three models, while the association between MSI and CD41–CD61 expression emerged only in model 3.

**Figure 4:**
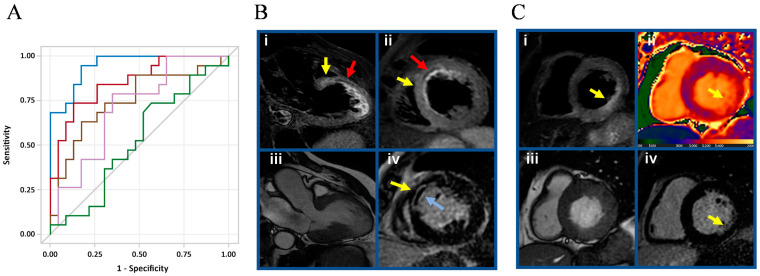
CD41–CD61 expression and size discriminated patients with MVO/MSI. (**A**) ROC analysis was used to evaluate the ability of sEV profile (concentration, dimension, and CD41–CD61 expression) to identify patients with MVO and/or low MSI. Violet line: CD41–CD61 expression; brown line: dimension; green line: concentration; red line: troponin peak; blue line: Model including all the variables of sEV profile and troponin. (**B**) Patient with unfavorable CMR and sEV profile. A 69-year-old man with acute anterior STEMI: Three-chamber long axis view (**i**) and mid ventricle short axis view (**ii**) T2-weighted triple inversion recovery (TIR T2) images showing myocardial edema (yellow arrow) and myocardial hemorrhage (red arrow) at anteroseptal wall. (**iii**) Three-chamber long axis view cine image at end-systole, showing akinesia at mid-to-apical anteroseptal wall and apical inferolateral wall. (**iv**) Mid ventricle short axis view late gadolinium enhancement (LGE) image showing transmural enhancement (yellow arrow) and microvascular obstruction (light blue arrow) at anteroseptal wall. (**C**) Patient with favorable CMR and sEV profile. A 50-year-old man with acute inferolateral STEMI. (**i**) Basal ventricle short axis view TIR T2 image showing myocardial edema (yellow arrow) at inferolateral wall. (**ii**) Basal ventricle short axis view native T1 mapping image showing focal increased T1 values (up to 1372 msec; values at remote myocardium 1004 ± 30 msec) in line with focal myocardial damage (yellow arrow) at inferolateral wall. (**iii**) Basal ventricle short axis view cine image at end-systole, showing mild hypokinesia at inferolateral wall. (**iv**) Basal ventricle short axis view LGE image showing subendocardial enhancement involving 25–50% wall thickness (yellow arrow).

Moreover, CD41–CD61 expression and size significantly discriminated patients with MVO/MSI in ROC curve analysis, with areas under the curve (AUC) ranging from 0.71 to 0.75 (Table S8). Of note, the sEV profile was able to increase the predictive ability of the troponin peak with an AUC value passing from 0.84 to 0.95 (Table S8, Figure 4A). Figure 4 shows representative cases of STEMI in patients with unfavorable (Figure 4B) and favorable (Figure 4C) CMR and sEV characteristics.

## 4. Discussion

The present study provides evidence for the first time that the signature of circulating sEVs reflects myocardial injury and CMR most important features in post-STEMI patients. Indeed, circulating sEV levels were higher in patients with anterior STEMI, LAD occluded artery and longer ischemic period. Moreover, the sEV size and expression of the platelet marker CD41–CD61, likely reflecting the level of circulating platelet-derived sEVs, were independent predictors of MVO and low MSI that are both predictors of short-term prognosis of acute STEMI after pPCI treatment and are key variables for risk-stratification of patients after STEMI [22].

CMR is an excellent tool for detecting prognostically relevant tissue damage such as MVO, with high accuracy and an excellent prediction of adverse remodeling and death in post-STEMI patients [23]. CMR is actually the technique of choice for the evaluation of post-infarction damage but it is not always applicable due to availability reasons or patients’ contraindications (e.g., claustrophobia, implanted electronic devices, etc.).

Currently, the TIMI risk score is used to predict MVO and prognosis in clinical practice [24], but its sensitivity in recognizing advanced tissue damage is far from being desirable, as often it underestimates the prevalence of MVO compared to CMR [25,26]. Therefore, it would be helpful to identify additional early and easy measurable determinants of myocardial injury is crucial for adequate prognostication in this specific clinical context. Circulating sEVs have been proposed as ideal markers for pathological conditions because they fulfill many of the properties/characteristics of biomarkers: easy and rapid detection and great stability. Moreover, sEV release and composition faithfully reflect the presence of activated cells in vivo, including cells related to the cardiovascular system (e.g., platelets, endothelial/vascular cells, cardiomyocytes), and they are able to affect both physiological and pathological processes, such as immune responses, angiogenesis and wound healing.

The number of circulating sEVs has been demonstrated to change in several pathologies, including cardiovascular diseases. In particular, levels of sEVs are increased in patients undergoing CABG [27] and in acute STEMI compared to CCS [5], strongly suggesting that the blood sEV concentration, per se, may reflect the undergoing ischemic disease.

Consistent with these previous observations, in our study population, the sEVs number is altered according to some important clinical parameters, which are STEMI location, culprit coronary, and reperfusion time. 

The location of AMI is an important prognostic factor for the risk stratification of STEMI patients. Anterior wall infarction is associated with a worse prognosis (more extensive LV dysfunction and remodeling, heart failure, and higher mortality) compared to other STEMI locations [28,29].

We showed an increased number of circulating sEVs associated with anterior STEMI compared to a non-anterior counterpart. Accordingly, since anterior AMI is mainly caused by an occlusion of LAD, the increase in EVs is also detected when the culprit lesion is located in LAD. These higher levels of sEVs detected in anterior myocardial infarction likely result from the larger amount of irreversible ischemic LV damage usually associated with anterior infarcts [30].

Moreover, the elevated number of sEVs found in late-presenting STEMI is likely due to the prolonged ischemic period suffered by these patients. Indeed, hypoxia stress has been demonstrated to be a stronger inducer of sEV release [31].

Interestingly, the number of sEVs was not altered by the extent of LGE and presence and extent of MVO, nor was it associated with MSI values; however, all these parameters were related to sEV size. We and others have already demonstrated that also sEV size can hold promising diagnostic and prognostic applications [5,32]. In this work, the dimension of sEVs is associated with CMR parameters: sEV size negatively correlated with MVO extent and patients with MVO and low MSI (<0.5) are characterized by sEVs with significantly smaller sEV dimension. The mechanisms governing the release, dimension and composition of sEVs are wide, and since we did not investigate these aspects we can only speculate that the difference in size observed may be driven by different cell types involved in the release of sEVs or by some modification in the release mechanisms.

By evaluating the expression of surface markers, we found that the main sources of sEVs in post-STEMI patients are platelets, leukocytes and endothelial cells. This expression pattern reflects cells mainly involved/activated during myocardial infarction and the biological processes relevant in the context of AMI, such as platelet and endothelial activation, immune regulation, cell–cell adhesion, and extracellular matrix regulation. Overall, the most highly expressed surface markers are those derived from platelets, including proteins related to platelet degranulation, activation, signaling and aggregation. We have recently shown that the expression of platelet markers on sEVs distinguished acute STEMI from CCS patients [5], showing their ability to reflect the undergoing platelet activation in acute STEMI. However, platelets are not only key players in primary hemostasis and thrombosis, but also contribute to many other pathophysiological processes, including wound healing and cardiac regeneration, through the release of growth factors, cytokines, and EVs.

Interestingly, anterior STEMI and MVO, robust predictors of adverse remodeling and poor outcome, were characterized by lower expression of the platelet marker CD41–CD61 and thus of platelet-derived sEVs. These data, in agreement with the observation of Gąsecka et al. showing an association between a lower plasma concentration of platelet-EVs and adverse remodeling at six months after AMI [6], strongly suggest that sEVs derived from platelets could be potentially implicated in the modulation of the post-STEMI reparative response to injury, with prognostic implications. Several studies have shown that platelets have a strong cardioprotective potential after ischemia and the mechanisms involved seem to depend on factors released by platelets, including EVs [8]. Indeed, EVs released from platelets carry several proteins and growth factors able to promote tissue survival and regeneration [33,34]. Therefore, a reduction in platelet-derived vesicles can negatively affect the recovery of the myocardium.

Sex-related factors influence the clinical manifestations and outcomes of cardiovascular disease and are now considered a new factor to be taken into account in disease diagnosis and prognosis [35]. In our study, none of the sEV characteristics differed between the sexes, suggesting that these sEV profiles can be reliable for both males and females. However, further investigations on a larger group are needed to confirm this statement. 

Finally, sEV size and CD41–CD61 were independent predictors of MVO/MSI and these sEV variables displayed a good predictive ability to discriminate patients with MVO/MSI. Of note, all variables here analyzed (sEV number, dimension and CD41–CD61 expression) are also able to increase the total predictive ability of troponin, which is one of the strongest values associated with clinical outcome, further suggesting that the sEV profile may be indicative of myocardial damage as detected by CMR.

The role of EVs in cardiovascular disease, and many of their intriguing properties provide the basis for extending EV analysis beyond basic research and into the clinical context. There are a growing number of studies showing that sEVs from blood samples are good candidates as part of a diagnostic/prognostic platform [36]. Accordingly, we and others have shown that either a single or a panel of differentially expressed sEV characteristics can significantly identify cardiovascular patients and also aid in risk stratification [5,6,27]. Overall, the analysis of circulating sEVs offers several advantages as potential biomarkers, as they are highly specific and sensitive, can be isolated from easily accessible biofluids, and contain a plethora of clinically relevant molecules that have a longer half-life compared to freely circulating counterparts, since the sEV membrane protects them from degradation [4]. In the clinical context here analyzed, sEVs could represent a faster and more cost-saving prognostic tool compared to CMR.

With this preliminary study, we suggested a panel of three sEV parameters for myocardial damage that have potential utility for clinical applications as prognostic tools. The analysis of EVs could accordingly be combined with biochemical, clinical and, if available, imaging data, defining a new strategy for the timely identification of high-risk patients and resulting in a patient-optimized treatment. Moreover, understanding the biological significance of platelet sEVs after STEMI may lead to the development of a new therapeutic strategy. In addition, sEVs may provide new insights into the underlying pathophysiological mechanism thus helping to evaluate new/different strategies to tailor an approach to alleviate myocardial damage.

Our results should be interpreted in the light of their limitations. First, our results are based on a relatively small sample size and should be considered as a hypothesis. Replication of these data and specific prospective studies with a larger sample size are needed to confirm the results of the current study. Second, systematic follow-up with CMR was not performed. Moreover, an evaluation of the clinical outcome (LV remodeling, heart failure incidence, myocardial infarction, etc.) at follow-up could be very useful to test the clinical implications of our data.

## 5. Conclusions

We reported for the first time the ability of sEVs isolated a few days after STEMI to reflect myocardial damage. After a careful validation of our data in a larger validation group, sEVs characterization may be potentially used as a tool for risk stratification in patients with STEMI.

Further characterization of sEVs is necessary to create a deeper pathophysiological understanding and to elucidate their possible role as a diagnostic tool and possibly also as a therapeutic target. 

## Figures and Tables

**Table 1:** Baseline Characteristics of the Study Population.

Baseline Characteristics	
Age, years	63 ± 9.69
Male, n (%)	31 (73.8)
Body mass index, Kg/m^2^	25.9 ± 3.7
Hypertension, n (%)	22 (52.4)
Smoking, n (%)	25 (59.2)
Hyperlipidemia, n (%)	13 (30.9)
Diabetes, n (%)	4 (9.5)
Family history of CAD, n (%)	10 (23.8)
Peak Troponin I, ng/dL	22.19 (11.2–32.4)
Time to pPCI, h	2.25 (2–4)
**Site of myocardial infarction**	
Anterior STEMI, n (%)	19 (45.2)

Values are mean ± SD, n (%), or median (confidence interval). CAD: coronary artery disease, pPCI: Primary percutaneous coronary intervention.

**Table 2:** Information about CMR characteristics.

CMR Characteristics	
Days after pPCI	5 ± 3
LVEDVi, mL/m^2^	86.5 ± 21.3
LVESVi, mL/m^2^	43.1 ± 22.4
LVEF, %	51 ± 11.2
LV mass, g	111.9 ± 26.1
RVEDVi, mL/m^2^	67.1 ± 14.3
RVESVi, mL/m^2^	26.3 ± 8.8
RVEF, %	61.2 ± 8.2
AAR, g	20.5 (12–31.6)
AAR, % LV mass	18.8 (12.6–29.4)
LGE mass, g	4.25 (3.2–18.7)
LGE, % LV mass	5.2 (2.5–15.7)
MVO, prevalence	20 (47.6)
MVO mass, g	2.7 ± 4.8
MVO mass, % LV mass	2.8 ± 4.9
MVO mass, % LGE mass	14.8 ± 30
MSI	0.43 ± 0.39

Values are mean ± SD, median (confidence interval), or n (%). AAR: area at risk, LGE: late gadolinium enhancement, LV: left ventricular, LVEDVi: indexed left end-diastolic volume, LVEF: left ventricular ejection fraction, LVESVi: indexed left end-systolic volume, MVO: microvascular obstruction, MSI: myocardial salvage index, RVEDVi: indexed right end-diastolic volume, RVESVi: indexed right end-systolic volume, RVEF: indexed right end.

**Table 3:** Associations between variables of sEV profile and reperfusion myocardial injury (MVO and MSI) analyzed by univariate and multivariate logistic models.

	Model 1	Model 2	Model 3
	Associations between sEV parameters and MSI and/or MVO		
Concentration	1.00 (1.00–1.00)	1.00 (1.00–1.00)	1.00 (1.00–1.00)
Size	**0.94 (0.89–0.99)**	**0.93 (0.88–0.99)**	**0.93 (0.87–0.98)**
CD41–CD61	**0.15 (0.02–0.88)**	**0.13 (0.02–0.97)**	**0.04 (0–0.61)**
	**Associations between sEV parameters and MVO**		
Concentration	1.00 (1.00–1.00)	1.00 (1.00–1.00)	1.00 (1.00–1.00)
Size	**0.93 (0.88–0.98)**	**0.93 (0.87–0.98)**	**0.91 (0.85–0.97)**
CD41–CD61	**0.16 (0.03–0.92)**	**0.12 (0.02–0.92)**	**0.03 (0–0.55)**
	**Associations between sEV parameters and MSI**		
Concentration	1.00 (1.00–1.00)	1.00 (1.00–1.00)	1.00 (1.00–1.00)
Size	0.96 (0.91–1)	0.96 (0.91–1)	0.94 (0.89–1)
CD41–CD61	0.29 (0.06–1.47)	0.14 (0.02–1.07)	**0.07 (0.01–0.74)**

Data are reported as odds ratio (95% confidence interval). In bold the statistically significant values. Model 1: unadjusted, model 2: adjusted to STEMI site; model 3: a model including all the three sEV profile variables and STEMI site.

## Data Availability

The data presented in this study are available on request from the corresponding author.

## Supplementary Materials

The following supporting information can be downloaded at: https://www.mdpi.com/article/10.3390/biom13101470/s1, Figure S1: Network graph summarizing pathways analysis results, performed using the expressed antigens; Table S1: Relationship between sEVs profile and relevant clinical and CMR features; Table S2: Correlations between sEV dimension/concentration and clinical/CMR parameters; Table S3: The relation between MACSPlex-antigens and the associated genes; Table S4: Details of pathway analysis; Table S5: Correlations between CD41–CD61 expression and clinical/CMR parameters; Table S6: sEV characteristics analyzed according to gender; Table S7: Correlation between sEV parameters and age of patients; Table S8: ROC analysis.
