# Supplementary material for: Circulating Small Extracellular Vesicles Reflect the Severity of Myocardial Damage in STEMI Patients
Source: Biomolecules. 2023 Sep 29;13(10):1470. doi: 10.3390/biom13101470 (PMC10605123; doi:10.3390/biom13101470)
Supplement: Supplementary file 1 [file biomolecules-13-01470-s001.zip › biomolecules-2567543-supplementary.pdf]

Circulating small extracellular vesicles reflect the severity of myocardial damage in STEMI patients.

Zarà et al.

Supplementary Tables

Table S1: Relationship between sEVs profile and relevant clinical and CMR features.

	**STEMI site: Anterior**	**STEMI site: Non-anterior**	*p value*
Number (particles/ml)	$4.7x10^{11} \pm 1.07x10^{11}$	$3.49x10^{11} \pm 9.82\ x10^{10}$	**0.0001**
Dimension (nm)	81.49 ± 13.94	80.97 ± 15.50	0.73
	**Culprit lesion: LAD**	**Culprit lesion: Other vessels**	
Number (particles/ml)	$4.4x10^{11} \pm 1.29x10^{11}$	$3.71x10^{11} \pm 9.77x10^{11}$	**0.045**
Dimension (nm)	81.92 ± 13.15	80.47 ± 16.33	0.49
	**Presentation time: Late**	**Presentation time: Early**	
Number (particles/ml)	$4.4\ x10^{11} \pm 1.13x10^{11}$	$3.79\ x10^{11} \pm 1.18x10^{11}$	**0.038**
Dimension (nm)	80.68 ± 13.59	81.63 ± 15.66	0.99
	**MSI ≤ 0.5**	**MSI > 0.5**	
Number (particles/ml)	$4.02\ x10^{11} \pm 1.24x10^{11}$	$4.14\ x10^{11} \pm 1.12x10^{11}$	0.59
Dimension (nm)	77.24 ± 13.44	88.10 ± 14.40	**0.014**
	**Presence of MVO**	**Absence of MVO**	
Number (particles/ml)	$4.1x10^{11} \pm 1.26x10^{11}$	$4.1x10^{11} \pm 1.17x10^{11}$	0.99
Dimension (nm)	74.24 ± 13.74	86.68 ± 13.27	**< 0.002**

sEVs number and size was evaluated by NTA after isolation from plasma collected 3-5 days post STEMI. Patients were divided according to STEMI site, culprit lesion artery, presentation time (Early < 3 hours, Late ≥ 3 hours), MVO presence, MSI (low < 0.5; high > 0.5). Mean ± SD are reported. In bold the statistically significant values.

Table S2: Correlations between sEV dimension/concentration and clinical/CMR parameters

	**sEV concentration**	**sEV dimension**
Peak Troponin	r= -0.12; *p*= 0.42	r= -0.20; *p*= 0.20
LVEDVi	r= 0.05; *p*= 0.75	r= -0.16; *p*= 0.33
LVESVi	r= 0.13; *p*= 0.40	r= -0.20; *p*= 0.21
LVEF	r= -0.14; *p*= 0.37	r= 0.18; *p*= 0.25
LV mass	r= 0.10; *p*= 0.54	r= 0.01; *p*= 0.96
RVEDVi	r= -0.11; *p*= 0.50	r= 0.01; *p*= 0.97
RVESVi	r= -0.11; *p*= 0.47	r= 0,17; *p*= 0.30
RVEF	r= -0.08; *p*= 0.60	r= -0,19; *p*= 0.23
AAR	r= 0.17; p= 0.27	r= -0,02; *p*= 0.92
AAR (% LV mass)	r= 0.20; *p*= 0.20	r= -0.06; *p*= 0.71
LGE mass	r= -0.08; *p*= 0.63	r= -0.37; *p*= 0.01
LGE (% LV mass)	r= -0.07; *p*= 0.65	r= -0.40; *p*= 0.01
MVO mass	r= 0.09; *p*= 0.55	r= -0.32; *p*= 0.04
MVO mass (% LV mass)	r= 0.08; *p*= 0.62	r= -0.33; *p*= 0.03
MVO mass (% LGE mass)	r= 0.07; *p*= 0.66	r= -0.39; *p*= 0.01
MSI	r= 0.09; *p*= 0.59	r= 0.13; *p*= 0.43

AAR: area at risk, CMR: cardiac magnetic resonance, LGE: late gadolinium enhancement, LV: left ventricular, LVEDVi: indexed left end-diastolic volume, LVEF: left ventricular ejection fraction, LVESVi: indexed left end-systolic volume, MVO: microvascular obstruction, MSI: myocardial salvage index, RVEDVi: indexed right end-diastolic volume, RVESVi: indexed right end-systolic volume, RVEF: indexed right end.

Table S3: The relation between MACSPlex-antigens and the associated genes.

Antigens	Genes
CD29	ITGB1
CD41b	ITGA2B
CD42a	GP9
CD9	CD9
HLA-ABC	HLA-A
CD81	CD81
CD56	NCAM1
CD62P	SELP
CD24	CD24
HLA-DRDPDQ	HLA-DRB1
CD31	PECAM1
CD45	PTPRC
CD40	CD40
CD3	CD3
CD44	CD44
CD25	IL2RA
CD2	CD2
CD8	CD8A
CD69	CD69
SSEA-4	FUT4
CD49e	ITGA5
CD146	MCAM
CD105	ENG
CD63	CD63
ROR1	ROR1
CD142	F3
CD20	MS4A1
CD11c	ITGAX
CD14	CD14
CD1-c	CD1C
CD4	CD4
CD209	CD209
CD86	CD86
CD133/1	PROM1
CD19	CD19

Gray antigens denote not expressed antigens.

Table S4. Details of pathway analysis.

ID	Group	Term	Term p-value	% Associated Genes	Nr. Genes	Associated Genes Found
R-HSA:9013408	Cell-Cell interaction	RHOG GTPase cycle	0.008854	2.7	2	[ITGB1, MCAM]
R-HSA:373755	Cell-Cell interaction	Semaphorin interactions	0.00689	3.08	2	[ITGB1, PTPRC]
R-HSA:1566948	Cell-Cell interaction	Elastic fibre formation	0.0033565	4.44	2	[ITGA5, ITGB1]
R-HSA:76009	Platelet activation	Platelet Aggregation (Plug Formation)	0.0025299	5.13	2	[GP9, ITGA2B]
R-HSA:202427	Cell-Cell interaction	Phosphorylation of CD3 and TCR zeta chains	0.0008045	9.09	2	[HLA-DRB1, PTPRC]
R-HSA:416700	Cell-Cell interaction	Other semaphorin interactions	0.0005976	10.53	2	[ITGB1, PTPRC]
R-HSA:913531	Immunity	Interferon Signaling	0.0005135	2.01	4	[CD44, HLA-A, HLA-DRB1, NCAM1]
R-HSA:3000178	Cell-Cell interaction	ECM proteoglycans	0.0004006	3.95	3	[ITGA2B, ITGB1, NCAM1]
R-HSA:75892	Platelet activation	Platelet Adhesion to exposed collagen	0.0003687	13.33	2	[GP9, ITGB1]
R-HSA:1566977	Cell-Cell interaction	Fibronectin matrix formation	0.0000532	33.33	2	[ITGA5, ITGB1]
R-HSA:877300	Immunity	Interferon gamma signaling	0.0000248	4.4	4	[CD44, HLA-A, HLA-DRB1, NCAM1]
R-HSA:76002	Platelet activation	Platelet activation, signaling and aggregation	0.0000076	2.28	6	[CD63, CD9, GP9, ITGA2B, PECAM1, SELP]
R-HSA:76005	Platelet activation	Response to elevated platelet cytosolic Ca2+	0.0000046	3.73	5	[CD63, CD9, ITGA2B, PECAM1, SELP]
R-HSA:198933	Immunity	Immunoregulatory interactions between Lymphoid and	0.0000043	3.79	5	[CD40, CD81, CD8A, HLA-A, ITGB1]

		non-Lymphoid cells				
R-HSA:114608	Platelet activation	Platelet degranulation	0.0000038	3.88	5	[CD63, CD9, ITGA2B, PECAM1, SELP]
R-HSA:373760	Cell-Cell interaction	L1CAM interactions	0.0000026	4.2	5	[CD24, ITGA2B, ITGA5, ITGB1, NCAM1]
R-HSA:216083	Cell-Cell interaction	Integrin cell surface interactions	0.0000005	5.88	5	[CD44, ITGA2B, ITGA5, ITGB1, PECAM1]
R-HSA:202733	Cell-Cell interaction	Cell surface interactions at the vascular wall	0.0000002	4.38	6	[CD2, CD44, ITGA5, ITGB1, PECAM1, SELP]
R-HSA:445144	Cell-Cell interaction	Signal transduction by L1	0.0000001	19.05	4	[ITGA2B, ITGA5, ITGB1, NCAM1]

Table S5: Correlations between CD41-CD61 expression and clinical/CMR parameters

	**CD41-CD61 expression**
Peak Troponin	r= -0.09; *p*= 0.55
LVEDVi	r= -0.03; *p*= 0.83
LVESVi	r= -0.15; *p*= 0.34
LVEF	r= 0.31; *p*= 0.04
LV mass	r= 0.05; *p*= 0.76
RVEDVi	r= 0.16; *p*= 0.32
RVESVi	r= 0.01; *p*= 0.94
RVEF	r= 0.19; *p*= 0.22
AAR	r= -0.18; p= 0.25
AAR (% LV mass)	r= -0.21; *p*= 0.19
LGE mass	r= -0.20; *p*= 0.19
LGE (% LV mass)	r= -0.20; *p*= 0.20
MVO mass	r= -0.40; *p*= 0.01
MVO mass (% LV mass)	r= -0.39; *p*= 0.01
MVO mass (% LGE mass)	r= -0.35; *p*= 0.02
MSI	r= 0.19; *p*= 0.24

AAR: area at risk, CMR: cardiac magnetic resonance, LGE: late gadolinium enhancement, LV: left ventricular, LVEDVi: indexed left end-diastolic volume, LVEF: left ventricular ejection fraction, LVESVi: indexed left end-systolic volume, MVO: microvascular obstruction, MSI: myocardial salvage index, RVEDVi: indexed right end-diastolic volume, RVESVi: indexed right end-systolic volume, RVEF: indexed right end.

Table S6. sEV characteristics analyzed according to gender.

	**Male**	**Female**	*p*
Number (particles/ml)	$4.19 \times 10^{11} \pm 1.20 \times 10^{11}$	$3.76 \times 10^{11} \pm 1.19 \times 10^{11}$	0.27
Dimension (nm)	81.82 ± 15.58	76.86 ± 10.99	0.51
CD41-CD61 expression (ng/sEV)	9.2e-009 ± 8.2e-009	8.7e-009 ± 2.9e-009	0.41

Table S7. Correlation between sEV parameters and age of patients

	**r**	***p***
Number (particles/ml)	0.06	0.68
Dimension (nm)	-0.18	0.25
CD41-CD61 expression	-0.17	0.27

Table S8. ROC analysis

	AUC	95% CI
Concentration	0.53	0.34-0.70
Size	0.75	0.59-0.90
CD41-CD61	0.71	0.55-0.86
Peak Troponin	0.84	0.74-0.97
Model	0.95	0.89-1.00

AUC: area under the curve, CI: confidence interval

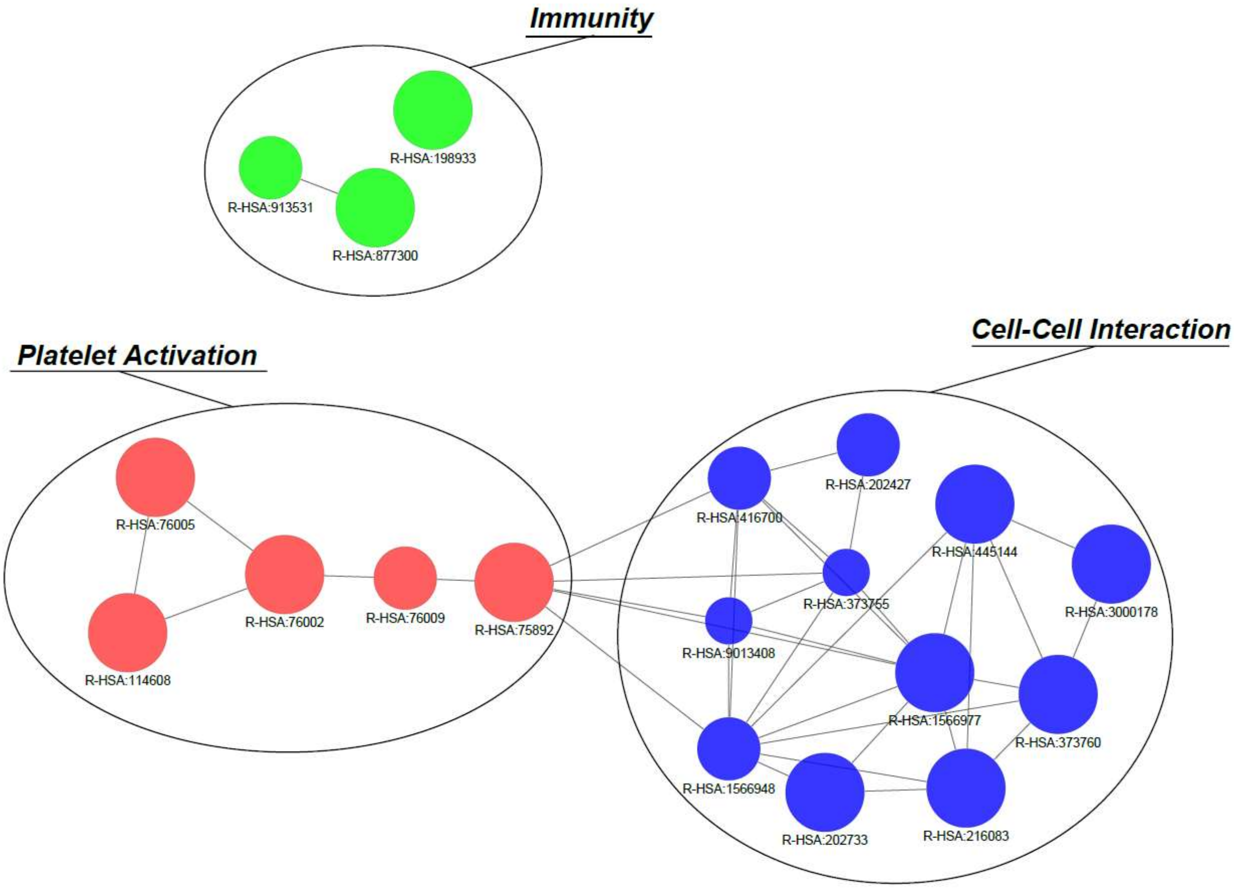

Figure S1: Network graph summarizing pathways analysis results, performed using the expressed antigens. Each node represents a specific Reactome pathway while the links thickness denotes the overlapping degree (k-score) between nodes. The node size reflects the significance: the higher the node, the more significant the pathway.
